# Tear ATG5 as a Potential Novel Biomarker in the Diagnosis of Sjögren Syndrome

**DOI:** 10.3390/diagnostics11010071

**Published:** 2021-01-04

**Authors:** Yong-Soo Byun, Hyun Jung Lee, Soojung Shin, Moon Young Choi, Hyung-Seung Kim, So-Hyang Chung

**Affiliations:** 1 Department of Ophthalmology and Visual Science, Catholic Institute for Visual Science, Seoul St. Mary’s Hospital, College of Medicine, The Catholic University of Korea, Seoul 06591, Korea; mdbyun@catholic.ac.kr (Y.-S.B.); amgine13@hotmail.com (S.S.); momotidae@gmail.com (M.Y.C.); sara514@catholic.ac.kr (H.-S.K.); 2 Department of Biochemical Engineering, Seoul University, Seoul 02192, Korea; hyunjung1203@gmail.com

**Keywords:** autophagy, ATG5, biomarkers, dry eye, Sjögren syndrome

## Abstract

Autophagy has been suggested to have an important role in the pathogenesis of Sjögren syndrome (SS). We previously identified that autophagy related 5 (ATG5) was elevated in the tear and conjunctival epithelial cells of SS dry eyes (DE) compared to non-SS DE. The purpose of this study was to investigate the role of tear ATG5 as a potential biomarker in the diagnosis of SS. To confirm this hypothesis, we evaluated the tear ATG5 concentration, and other ocular tests (Schirmer I, tear breakup time (TBUT), ocular surface staining (OSS) score, ocular surface disease index (OSDI)) in SS and non-DE, and compared their diagnostic performance to discriminate SS from non-SS DE. Tear ATG5 showed the greatest area under the curve (AUC = 0.984; 95% CI, 0.930 to 0.999) among the tests, and a 94.6% sensitivity and 93.6% specificity at a cutoff value of >4.0 ng/mL/μg. Our data demonstrated that tear ATG5 may be helpful as an ocular biomarker to diagnose and assess SS. In the future, the diagnostic power of tear ATG for SS should be validated.

## 1. Introduction

Sjögren syndrome (SS) is a systemic autoimmune disorder that affects the exocrine glands such as lacrimal and salivary glands, resulting in dry eye and dry mouth. There has been little disagreement among rheumatologists about the diagnosis of SS in a patient with obvious findings on the physical examination of dry eye, dry mouth, and the presence of serologic markers. Recently, the American College of Rheumatology/European League Against Rheumatism (ACR/EULAR) defined the diagnostic criteria, leading to consensus in research and clinical trial reports [1].

Despite extensive studies over the past decade, the pathogenesis of SS has not been clearly understood. In general, it has been widely known that overexpression of inflammatory cytokines and lymphocyte infiltration with T and NK cells was observed in the affected glands, where secondary B-cell activation and autoantibody production is induced [2]. In addition, environmental trigger and genetic predisposition are thought to contribute to the development of SS [3].

Autophagy has been suggested to have a certain role in the pathogenesis of SS, although data to support this role are limited [4,5,6]. Autophagy is a self-eating process that maintains cellular homeostasis through lysosome-mediated degradation and recycling of cellular components and organelles. In addition to its inherent function against cellular stress, autophagy has also been implicated in the pathogenesis of autoimmune diseases [7]. Enhanced autophagy is thought to be involved in SS-A and SS-B redistribution on the cellular surface and subsequent production of autoantibodies in secretory epithelial cells of the salivary glands [8,9,10]. Our recent study also reported that autophagy markers were elevated in the tear and conjunctival epithelial cells of patients with SS dry eye (DE), unlike non-SS DE [11]. Autophagy related 5 (ATG5) is known as a key protein involved in the elongation of the phagophoric membrane, reflecting the increase or decrease of autophagy reaction.

The Schirmer I test and ocular surface staining (OSS) are currently used as ocular tests for diagnosing SS along with serologic tests and oral tests [12,13]. Due to the high variability and measurement error of these tests, however, researchers have been trying to develop more specific ocular biomarkers or tests for SS [14,15,16,17]. For this reason, we evaluated the diagnostic performance of tear autophagy related 5 (ATG5) for discriminating SS DE and compared it with other dry eye parameters included in diagnostic criteria of SS (Schirmer I and OSS).

## 2. Materials and Methods

This retrospective observational study was conducted at the Department of Ophthalmology at Seoul St. Mary’s Hospital, the Catholic University of Korea. The study protocol was determined according to the guidelines of the Declaration of Helsinki and approved by Seoul St. Mary’s Hospital Institutional Review Board (protocol code, KC13ONMI0646; date on approval, 1 October 2013), and informed consent was obtained from all subjects. We reviewed patients with aqueous deficient-type DE who visited the Ophthalmology Clinic, Seoul St. Mary’s Hospital, from 2015 to 2018. The aqueous deficient-type DE was defined as subjects having ocular symptom (ocular surface disease index (OSDI) score ≥13) and Schirmer I value ≤10 mm/5 min, or tear breakup time (TBUT) ≤less than 10 s [18]. All subjects were referred to the Rheumatology Clinic for SS work-up. Serology tests for anti-SS-A, and anti-SSB antibody, and other autoantibodies were performed at the Rheumatology Clinic, and labial salivary gland biopsy was selectively conducted in undetermined patients with negative serologic result in the Otorhinolaryngology Clinic. The definite diagnosis of SS was made according to the diagnostic criteria of 2016 ACR/EULAR. The following conditions were excluded: allergic conjunctivitis, lid abnormalities, any ocular surgeries or contact lens usage within last 6 months, punctual plug, meibomian gland dysfunction above grade 2, and subjects taking systemic medications, and any topical treatments other than artificial tears within the last 3 months.

Parameters related to DE, Schirmer I test, TBUT, OSS score, and OSDI were measured. The OSS score was determined according to the Sjögren’s International Collaborative Clinical Alliance registry ocular examination protocol [12]. Briefly, the OSS score ranged from 0 to 12, which was summed with cornea staining score (0 to 6) and temporal and nasal conjunctival score (0 to 3, respectively). Corneal staining was evaluated by slit-lamp examination after fluorescein dye was administered. The corneal staining score was measured as follows: a score of 0, if no punctate epithelial erosions (PEEs) were observed; 1, if 1–5 PEEs; 2, if 6–30 PEEs; and 3, if more than 30 PEEs. In the case of more than one patch that could be seen of confluent staining, pupillary area of the cornea, or filament anywhere on the cornea that was stained, an additional point was calculated for each. The conjunctival staining score was determined separately in the temporal and nasal bulbar conjunctiva after 1% Lissamine green dye (Leiter’s Pharmacy, San Jose, CA, USA) was applied. A score of 0 was appointed for 0 to 9 stained dots; 1 for 10 to 32 dots; 2 for 33 to 100 dots; and 3 for more than 100 dots.

The tear ATG5 concentration was measured using the Schirmer’s strips according to the protocol previously reported [19]. Briefly, cryopreserved strips were individually placed into tubes containing extraction buffer (phosphate-buffered saline with 0.5 M NaCl and 0.5% Tween-20). After 1 h, the buffer with strip was transferred into a 1.5 mL tube equipped with a mini cell strainer and centrifuged at 12,000 rpm for 15 min. Total protein amount (ng/mL) in collected fluid was quantified using the BCA Protein Assay kit (Thermo Fisher Scientific, Rockford, IL, USA). The ATG5 concentration (ng/mL) in collected fluid was measured using a commercial ELISA kit (MBS2025053, Human ATG5 ELISA kit, MyBioSource, San Diego, CA, USA) and the relative ratio of ATG5 to total protein (ng/mL/μg) was calculated. Data from the eyes with the higher OSS score were collected for analysis; if both eyes had the same score, the right eye was included.

Statistical analysis was performed with SPSS version 15.0 (SPSS Inc., Chicago, IL, USA). After data normality was determined using the D’Agostino–Pearson omnibus test, the Mann–Whitney U test and the Spearman rank correlation test were performed to compare two groups (SS and non-SS DE group) and assess the correlation between variables, respectively. The receiver operating characteristic (ROC) curves were made using MedCalc Statistical Software version 15 (MedCalc Software bv, Ostend, Belgium). The descriptive data were expressed as the mean ± SD with median and range. A two-sided *p*-value < 0.05 was considered statistically significant.

## 3. Results

The present study enrolled 55 patients with SS DE and 31 patients with non-SS DE. The SS DE group showed significantly younger age (mean ± standard deviation (SD), 50.7 ± 10.6 vs. 55.7 ± 11.1), shorter tear breakup time (TBUT, 3.0 ± 2.3 vs. 4.0 ± 1.6), higher score in OSS (4.7 ± 3.0 vs. 2.5 ± 1.5) and conjunctival staining (2.6 ± 1.9 vs. 0.6 ± 0.9), and higher tear ATG5, compared to the non-SS DE group (Table 1). Female predominance, Schirmer I value, cornea staining score, and ocular surface disease index (OSDI) score were not different between the two groups.

In the SS DE group, tear ATG5 showed a positive correlation with OSS (r = 0.3898; 95% confidence interval (CI) = 0.1309 to 0.5989), cornea (0.2833; 0.01132 to 0.5162), and conjunctiva staining score (0.3687; 0.1066 to 0.5829), but the correlations between tear ATG5 and those variables were not strong (Table 2). In contrast, there was no statistically significant correlation between tear ATG5 and other ocular tests in the non-SS DE group (Table 3).

**Figure 1:**
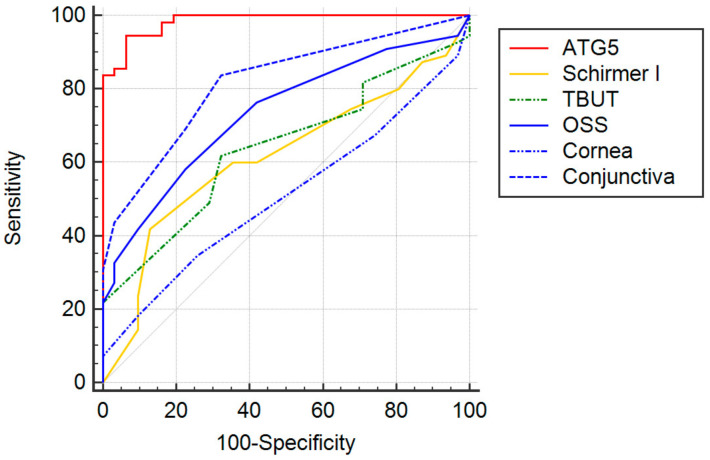
Receiver operating characteristic (ROC) curves for different parameters. The greatest area under the curve (AUC) was found for tear ATG5 (0.984, 95% confidence interval (CI), 0.930 to 0.999), followed by the conjunctiva staining score (0.817; 95% CI, 0.719 to 0.892), the OSS score (0.734; 95% CI, 0.628 to 0.824), the TBUT (0.640; 95% CI, 0.529 to 0.741), the Schirmer I test (0.616; 95% CI, 0.505 to 0.719), and the cornea staining score (0.508; 95% CI, 0.398 to 0.618).

The diagnostic performances of tear ATG5 and dry eye parameter tests were compared with a receiver operating characteristic (ROC) curve (Figure 1). Tear ATG5 demonstrated the greatest area under the curve (AUC = 0.984; 95% CI, 0.930 to 0.999), followed by the conjunctiva staining score (0.817; 0.719 to 0.892), the OSS score (0.734; 0.628 to 0.824), the TBUT (0.640; 0.529 to 0.741), the Schirmer I test (0.616; 0.505 to 0.719), and the cornea staining score (0.508; 0.398 to 0.618).

Tear ATG5 showed a 94.6% sensitivity and 93.6% specificity at a cutoff value of >4.0 ng/mL/μg (Table 4). The Schirmer I value had a 74.6% sensitivity and 32.3% specificity at a cutoff value of ≤5 mm; TBUT, a 61.8% sensitivity and 67.7% specificity at a cutoff value of ≤4 s; the OSS score, a 58.2% sensitivity and 77.4% specificity at a cutoff value of ≥3; and the OSS score, a 32.7% sensitivity and 96.8% specificity at a cutoff of ≥5.

## 4. Discussion

Our data showed that tear ATG5 elevates significantly in SS DE and it has a better diagnostic performance to discriminate SS DE from non-SS DE compared to other ocular tests used currently for a diagnosis of SS. Tear ATG5 at a cutoff of >4.0 ng/mL/μg had a positive likelihood ratio (LR) of 14.7 with a 94.6% sensitivity and 93.6% specificity for SS in patients with aqueous deficient-type DE, which was greater than the values of the Schirmer I test, TBUT, and the OSS score.

Additionally, tear ATG5 showed a positive correlation with the OSS score especially in SS DE patients, and a stronger correlation with conjunctiva staining score when analyzed separately for conjunctiva and cornea staining in OSS. This is in line with previous studies, in which conjunctival staining score correlated well with various inflammatory cytokines in SS [17,20]. They demonstrated that conjunctival staining scores significantly correlated with the expression of IFN-γ, IL-6, IL-17, and MMP-9 in both DE groups. Interestingly, correlation coefficients of all cytokines were much higher in SS DE compared to non-SS DE [21].

Schirmer I and TBUT are simple and validated tests performed to evaluate DE. However, they are not used alone in practice to diagnose or assess DE due to their high variability and measurement error [14,15,16]. Versura et al. [22] reported poor diagnostic performance of Schirmer I and TBUT as an ocular test for a diagnosis of SS and suggested to use them together with other examinations related to the ocular surface status. In their report, Schirmer I test at a cutoff of 5 mm/5 min had an LR of 1.75 with a 42% sensitivity and 76% specificity and TBUT at a cutoff of <10 s, an LR of 1.11 with an 82% sensitivity and 17% specificity. Our results correspond to their results that these conventional tests do not have good diagnostic performance for SS. Interestingly, TBUT showed a relatively greater AUC (0.640) than the Schirmer I test. This is believed to be because all subjects included in our study had TBUT of 10 or less, indicating the aqueous deficient-type DE. TBUT may be a better test to discriminate SS from non-SS DE than the Schirmer I test in subjects with aqueous deficient-type DE. In contrast, the OSS scoring system showed good diagnostic performance with an LR of 10.2 and 2.6 at a cutoff of ≥5 and ≥3, as previous studies reported [12]. However, there has been a controversy about the examiner’s bias for OSS scoring, although it has been accepted as the test of choice among many ocular tests for a diagnosis of SS [22].

Increasing bodies of evidence for inaccuracy of existing ocular tests has led to efforts to find reliable ocular biomarkers for a diagnosis of SS. Tear has been proposed as a good source of biomarkers for the diagnosis of SS [23]. Hamm-Alvarez indicated that tear cathepsin S activity was found to be significantly increased in patients with SS compared to patients with non-SS DE and healthy controls, and suggested that it may be a simple and non-invasive biomarker for the diagnosis and evaluation of SS [24]. However, they did not determine a cutoff value of tear cathepsin S to diagnose SS, so its diagnostic performance cannot be compared to other tests. Akpek et al. [17] reported that tear levels of IL-8, a highly neutrophil-specific chemoattractant, can potentially serve as a useful biomarker for differential diagnosis of SS DE from non-SS DE. They suggested that the elevation of IL-8 in SS may be associated with IL-17 and neutrophils, which are activated in chronic autoimmune response. Shinzawa et al. [25] reported that epidermal fatty acid-binding protein concentration significantly decreased in tears of patients with SS DE, and its level correlated with dry eye parameters. Recently, with the advances in tear analysis technology, it has become possible to simultaneously analyze various biomarkers in a small amount of tears, and a set of well-known biomarkers is expected to be used in the more accurate diagnosis of SS [26].

Autophagy plays important roles in redistributing the autoantigens SS-A and SS-B in epithelial cells of affected glands in SS, and autophagy dysregulation is also noted in lupus erythematous and rheumatoid arthritis [5,27,28]. ATG5 is specifically involved in the maturation of the autophagic membrane via conjugating with ATG12 during the autophagy process. ATG5 itself is thought to interfere with the maintenance of autoreactive T- and B-cell clones and to play a role in autophagy-mediated salivary homeostatic control in SS [4]. Our study demonstrated that tear ATG5 has a greater diagnostic performance for SS than other ocular tests, suggesting a potential biomarker of SS. The potential of tear ATG5 as a diagnostic marker for SS that we demonstrated in this study is only valid within the category of ocular tests for SS. No ocular test can replace the serology test or salivary test included in the diagnostic criteria of SS. If a novel ocular biomarker with a higher diagnostic performance for SS is discovered, it will be given a higher weighted score than other ocular tests in the diagnostic criteria of SS. This part should be verified through prospective study using a validation subject set.

Our research has some limitations. The present study was a retrospective observational research that analyzed ocular variables and tear ATG5 in patients who had already been evaluated for SS. Our follow-up study needs to confirm whether elevated ATG5 is consistent not only in tears but also in saliva and blood of SS patients, and verify the diagnostic power of tear ATG5 at a determined cutoff in the validation set, in order to use tear ATG5 as a biomarker in clinical practice. Second, the pathogenic association between elevation in tear ATG5 and SS has not been fully understood, although the pathogenic implication of autophagy in autoimmune disease including SS has been reported. In order to prove that ATG5 is a specific marker for SS, various methods including molecular research have to be performed in future studies.

In conclusion, we found that tear ATG5 was significantly higher in SS DE than non-SS DE, and it has very high sensitivity and specificity to differentiate SS DE from non-SS DE at a cutoff level of 4.0 ng/mL/μg. The diagnostic performance of tear ATG5 far exceeds that of other ocular tests that are currently used as diagnostic criteria for SS. In the future, the diagnostic power of tear ATG for SS should be verified in the validation set.

## Figures and Tables

**Table 1:** Comparison of descriptive data between Sjögren syndrome (SS) and non-Sjögren syndrome dry eye (DE) disease patients.

Parameters		Non-SS DE ^1^	SS DE ^2^	*p*-Value
Number of patients (eyes)		31	55	N/A
Sex	Male:Female	2:29	1:54	N/A
Age	Mean ± SD	55.7 ± 11.1	50.7 ± 10.6	0.0311
	Median, range	57, 21 to 77	51, 32 to 69	
Schirmer I	Mean ± SD	4.7 ± 2.5	3.7 ± 3.0	0.0721
	Median, range	5, 0 to 10	3, 0 to 10	
TBUT ^3^	Mean ± SD	4.0 ± 1.6	3.0 ± 2.3	0.0286
	Median, range	4, 2 to 7	3, 0 to 8	
OSS ^4^	Mean ± SD	2.5 ± 1.5	4.7 ± 3.0	0.0003
	Median, range	2, 0 to 7	4, 0 to 11	
Corneal staining	Mean ± SD	2.1 ± 1.0	2.2 ± 1.4	0.8996
	Median, range	2, 0 to 4	2, 0 to 5	
Conjunctival staining	Mean ± SD	0.6 ± 0.9	2.6 ± 1.9	<0.0001
	Median, range	0, 0 to 3	2, 0 to 6	
OSDI ^5^	Mean ± SD	30.4 ± 2.1	38.8 ± 20.6	0.0916
	Median, range	30.0, 56.8	36.6, 0 to 77	
Tear ATG5	Mean ± SD	2.48 ± 1.25	11.21 ± 6.06	<0.0001
	Median, range	2.58, 0.30 to 5.33	9.64, 3.47 to 25.30	

^1^ SS DE = Sjögren syndrome dry eye; ^2^ non-SS DE = non-Sjögren syndrome dry eye; ^3^ TBUT = tear breakup time; ^4^ OSS = ocular surface staining; ^5^ OSDI = ocular surface disease index. N/A = not applicable.

**Table 2:** Correlation of tear ATG5 with the Schirmer I, tear breakup time, ocular surface staining score, and ocular surface disease index in Sjögren syndrome dry eye disease patients.

	Spearman’s Rho	95% Confidence Interval	*p*-Value
Schirmer I	−0.08747	−0.3519 to 0.1899	0.5254
TBUT ^1^	−0.08178	−0.3469 to 0.1954	0.5528
OSS ^2^ score	0.3898	0.1309 to 0.5989	0.0033
Corneal score	0.2833	0.01132 to 0.5162	0.0361
Conjunctival score	0.3687	0.1066 to 0.5829	0.0056
OSDI ^3^ score	0.2087	−0.06801 to 0.4556	0.1263

^1^ TBUT = tear breakup time; ^2^ OSS = ocular surface staining; ^3^ OSDI = ocular surface disease index.

**Table 3:** Correlations of tear ATG5 with the Schirmer I, tear breakup time, ocular surface staining score, and ocular surface disease index in non-Sjögren syndrome dry eye disease patients.

	Spearman’s Rho	95% Confidence Interval	*p*-Value
Schirmer I	−0.1797	−0.5103 to 0.1972	0.3334
TBUT ^1^	−0.02740	−0.3875 to 0.3400	0.8837
OSS ^2^ score	−0.1460	−0.4843 to 0.2302	0.4331
Corneal score	−0.1141	−0.4590 to 0.2607	0.5412
Conjunctival score	−0.07130	−0.4243 to 0.3005	0.7031
OSDI ^3^ score	0.1143	−0.2605 to 0.4591	0.5405

^1^ TBUT = tear breakup time; ^2^ OSS = ocular surface staining; ^3^ OSDI = ocular surface disease index.

**Table 4:** Criterion values and coordinates of the ROC curve for tear ATG5 and ocular tests currently accepted.

Tests	Cutoff	Sensitivity (95% CI ^2^)	Specificity (95% CI ^2^)	Likelihood Ratio (95% CI ^2^)
Schirmer I	≤5 mm/5 min ^3^	74.6 (61.0 to 85.3)	32.3 (16.7 to 51.4)	1.1 (0.8 to 1.2)
TBUT	≤4 s	61.8 (47.7 to 74.6)	67.7 (48.6 to 83.3)	1.92 (1.1 to 3.3)
OSS ^1^	≥5 ^3^	32.7 (20.7 to 46.7)	96.8 (83.3 to 99.9)	10.2 (1.4 to 72.4)
OSS ^1^	≥3 ^4^	58.2 (44.1 to 71.3)	77.4 (58.9 to 90.4)	2.6 (1.3 to 5.1)
ATG5	>4.0 ng/mL/μg	94.6 (84.9 to 98.9)	93.6 (78.6 to 99.2)	14.7 (3.8 to 56.1)

^1^ OSS = ocular surface staining; ^2^ CI = confidence index; ^3^ proposed in the American College of Rheumatology/European League Against Rheumatism classification criteria for primary Sjögren syndrome [1]; ^4^ proposed in the Sjögren’s International Collaborative Clinical Alliance Cohort [20].

## Data Availability

The data presented in this study are available on request from the corresponding author.
